# Three-dimensional echo-planar cine imaging of cerebral blood supply using arterial spin labeling

**DOI:** 10.1007/s10334-016-0565-0

**Published:** 2016-05-25

**Authors:** Manoj Shrestha, Toralf Mildner, Torsten Schlumm, Scott Haile Robertson, Harald Möller

**Affiliations:** 1 Max Planck Institute for Human Cognitive and Brain Sciences, Stephanstraße 1a, 04103 Leipzig, Germany; 2 Center for In Vivo Microscopy, Duke University Medical Center, Durham, NC USA

**Keywords:** Echo-planar imaging, Cine magnetic resonance imaging, Perfusion imaging, Cerebral arteries

## Abstract

**Objective:**

Echo-planar imaging (EPI) with CYlindrical Center-out spatiaL Encoding (EPICYCLE) is introduced as a novel hybrid three-dimensional (3D) EPI technique. Its suitability for the tracking of a short bolus created by pseudo-continuous arterial spin labeling (pCASL) through the cerebral vasculature is demonstrated.

**Materials and methods:**

EPICYCLE acquires two-dimensional planes of *k*-space along center-out trajectories. These “spokes” are rotated from shot to shot about a common axis to encode a *k*-space cylinder. To track a bolus of labeled blood, the same subset of evenly distributed spokes is acquired in a cine fashion after a short period of pCASL. This process is repeated for all subsets to fill the whole 3D *k*-space of each time frame.

**Results:**

The passage of short pCASL boluses through the vasculature of a 3D imaging slab was successfully imaged using EPICYCLE. By choosing suitable sequence parameters, the impact of slab excitation on the bolus shape could be minimized. Parametric maps of signal amplitude, transit time, and bolus width reflected typical features of blood transport in large vessels.

**Conclusion:**

The EPICYCLE technique was successfully applied to track a short bolus of labeled arterial blood during its passage through the cerebral vasculature.

**Electronic supplementary material:**

The online version of this article (doi:10.1007/s10334-016-0565-0) contains supplementary material, which is available to authorized users.

## Introduction

Arterial spin labeling (ASL) has become a useful technique to study cerebral blood flow (CBF) in clinical and research applications [1]. Quantification of CBF by ASL is based on the difference between the brain tissue signal acquired in a labeled and in a control state. Arterial spin labeling in large vessels, although demonstrated very early [2], was initially of only minor interest and rather considered as a significant source of error. In recent years, however, ASL methods, such as dynamic spin labeling angiography [3], vessel-encoded dynamic magnetic resonance angiography (MRA) [4, 5] or mapping of arterial transit time (ATT) by intravascular signal selection [6], were developed in order to specifically assess the blood flow dynamics in large vessels. Increasing interest in those techniques is related to their potential to investigate collateral blood supply or time-dependent flow properties in selected vessels with potential applications to occlusive vascular disease [3, 5]. Provided that sufficient sensitivity and resolution are achieved, ASL-based techniques would yield this information without requiring exogenous contrast agents, ionizing radiation, or arterial catheterization—leading to intrinsic advantages over X-ray digital subtraction angiography (DSA). A separate general aspect is the need to characterize the tag delivery process sufficiently well for a reliable CBF quantification by ASL [7–9]. In this context, dynamic experiments may provide information on transit times and bolus dispersion [6].

Previous methods for ASL-based dynamic MRA [3–5] have used a segmented two-dimensional (2D) fast low-angle shot (FLASH) readout applied in a cine-like fashion in order to observe the inflow of labeled blood into the vasculature. The aim of the current work was to track a short bolus of labeled blood by a segmented three-dimensional (3D) echo-planar imaging (EPI) readout. This was performed to yield an isotropic coverage of the brain-feeding vessels, whereas it was not intended to obtain a distortion-free 2D slab of high spatial resolution.

In order to achieve a sufficient temporal resolution, 3D EPI has to be acquired in a segmented fashion. Between these segments, motion of the subject as well as phase differences will degrade the image quality. It is therefore advantageous to exploit sampling strategies, such as radial encoding, with an inherent oversampling of the central region of *k*-space [10, 11]. It is well known that this feature of radial encoding leads to a mitigation of motion artifacts [12, 13]. This immunity to motion has been exploited, for example, for high-resolution imaging of the lungs [14–16], cardiovascular imaging [17–19], or diffusion-weighted magnetic resonance imaging (MRI) [10, 20–22]. As another characteristic, each view samples the same amount of low and high spatial frequencies, which leads to advantageous undersampling properties [19, 23].

In this work, a novel sequence referred to as EPI with CYlindrical Center-out spatiaL Encoding (EPICYCLE) was developed. It was derived from a recent modification of 2D EPI achieving a very short echo time (*T*_E_) [24]. Therein, *k*-space is sampled by two center-out trajectories. An extension to three dimensions was achieved by rotating the center-out trajectory from shot to shot about the axis of a cylindrical section of *k*-space [25]. Thereby, a constant readout direction is maintained, and the phase-blips are rotated from shot to shot. To obtain a center-out trajectory, the phase-prephase gradient is omitted. We note that previous hybrid 3D EPI versions with cylindrical encoding have been implemented by rotating the readout gradient of a 2D EPI scheme while leaving the phase-blip and phase-prephase gradients unchanged [10, 11].

For cine imaging of cerebral blood supply, a combination with balanced pseudo-continuous ASL (pCASL) [26, 27] was used to create a short bolus of magnetically labeled water as endogenous tracer delivered by the brain-feeding arteries. The ASL bolus is tracked by continuous acquisition of the same segment of 3D *k*-space using EPICYCLE. An advantage over dynamic susceptibility contrast (DSC) MRI is that the pCASL experiment can be repeated almost without restrictions. The bolus is not created by an invasive intravenous injection of a contrast agent, but by the application of well-defined radiofrequency (RF) and gradient pulses, which yields a rectangular bolus shape at the labeling plane and excellent shot-to-shot reproducibility. The latter allows the acquisition of all segments in consecutive runs, which can then be combined to a full 3D *k*-space for each time step, yielding an effective temporal resolution equal to the acquisition time of a single segment, *T*_seg_. It should be noted that the above-stated dynamic MRA methods are also based on this principle [3–5].

A distinct difference in the current work was the use of shorter ASL bolus durations to permit the observation of the raising and falling edges of the vascular ‘brain response curve’. The influence of the (imaging) RF pulses on the shape of the ASL bolus was studied experimentally by varying *α* and theoretically by use of a kinetic model [5]. Under conditions where this influence is negligible, and by use of a reasonable voxel size, the recorded signal reflects the shape of the ‘arterial input function’ (AIF) in single voxels of the 3D imaging slab. Thus, ASL bolus tracking permits the direct observation of the downstream evolution of the AIF.

## Materials and methods

### Basic EPICYCLE pulse sequence

**Fig. 1:**
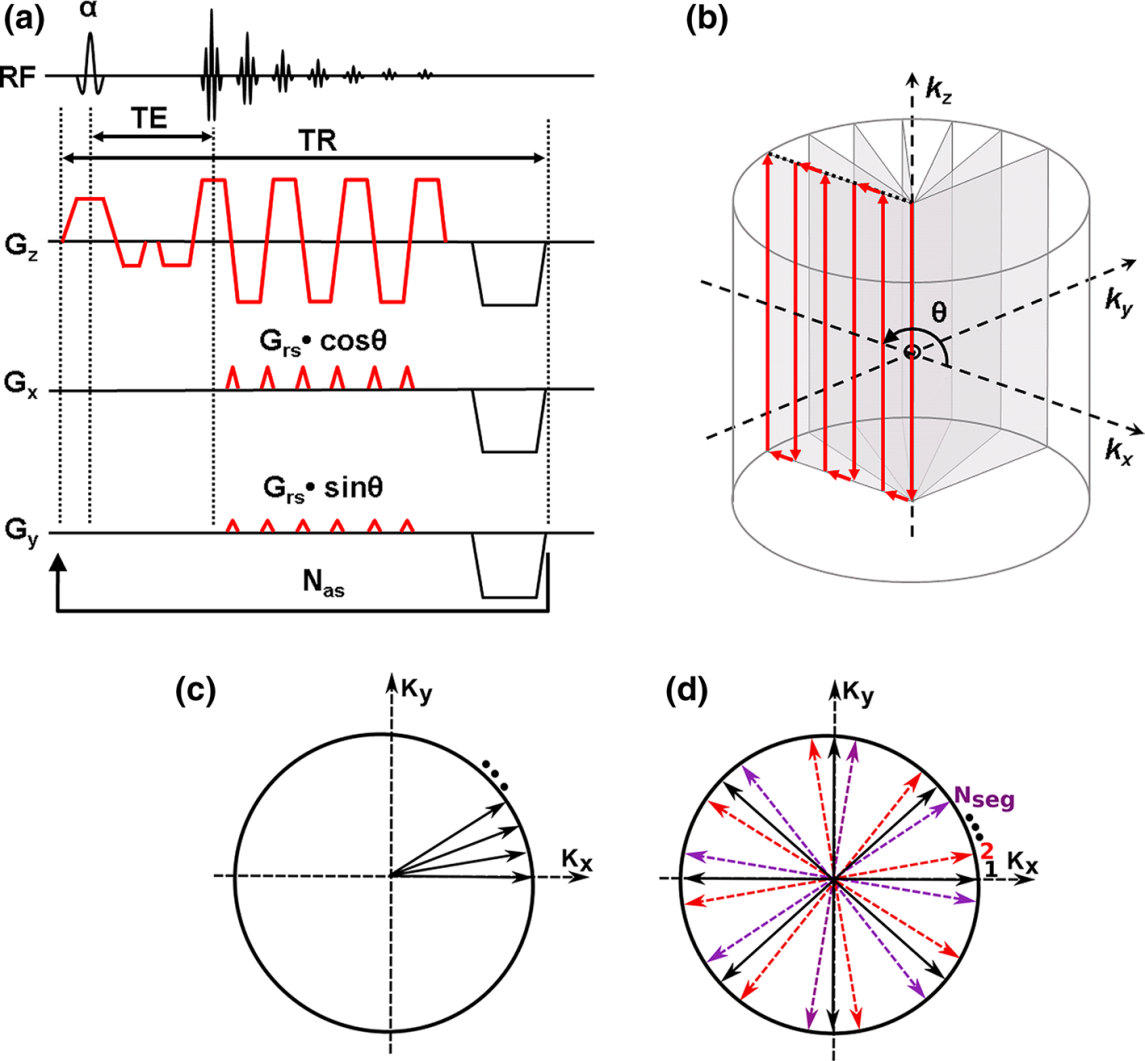
**a** EPICYCLE pulse sequence. In this example, slab-selection and slab-rephase gradients point along the read direction (here, the *z-axis*). Generally, their orientation can be adjusted arbitrarily, and their magnitude may be zero in case of a volume excitation. A spoiling gradient is applied along the diagonal through 3D *k*-space after the acquisition of each spoke to crush residual transverse magnetization. **b** Corresponding *k*-space trajectory. **c** Acquisition order of spokes for linear sampling and for **d** segmented sampling. Each arrow represents a trajectory in 3D *k*-space as in **b**. *Different colors* indicate different segments; *k*-space is filled in a way that all spokes from segment 1 (*black*) are acquired first, followed by all spokes from segment 2 (*red*), etc

Two half-planes of 2D *k*-space are acquired in a center-out fashion with opposite phase-blip polarities in a recent double-shot modification of EPI [24]. For EPICYCLE, this principle is expanded to cover a *k*-space volume; that is, a large number (*n*_*s*_ ≫ 2) of half-planes (i.e., spokes) are sampled along center-out trajectories with step-wise rotation of the phase-blip gradients about the readout direction (Fig. 1b). This encodes a cylindrical volume on a radial grid in the *k*_*x*_ *k*_*y*_-plane and on a Cartesian grid along the *k*_*z*_-axis. All spokes are acquired following slab-selective excitation with flip angle α and share one common line through the center of *k*-space along the *k*_*z*_- axis. These repetitively acquired central lines can be used to correct for motion induced by pulsatile flow along the *z*-axis or for drifts of the magnetic field [28].

Some auxiliary elements of the pulse sequence loop are not shown in Fig. 1a. Firstly, a volume-selective fat saturation RF pulse embedded by spoiling gradients is applied immediately before each excitation. Secondly, a sufficient number of dummy-loop passes are performed prior to data acquisition in order to establish a steady state of the magnetization. Subsequently, a template scan is recorded with all phase-blip gradients switched off and used for the correction of Nyquist ghosting artifacts [24, 29].

**Fig. 2:**
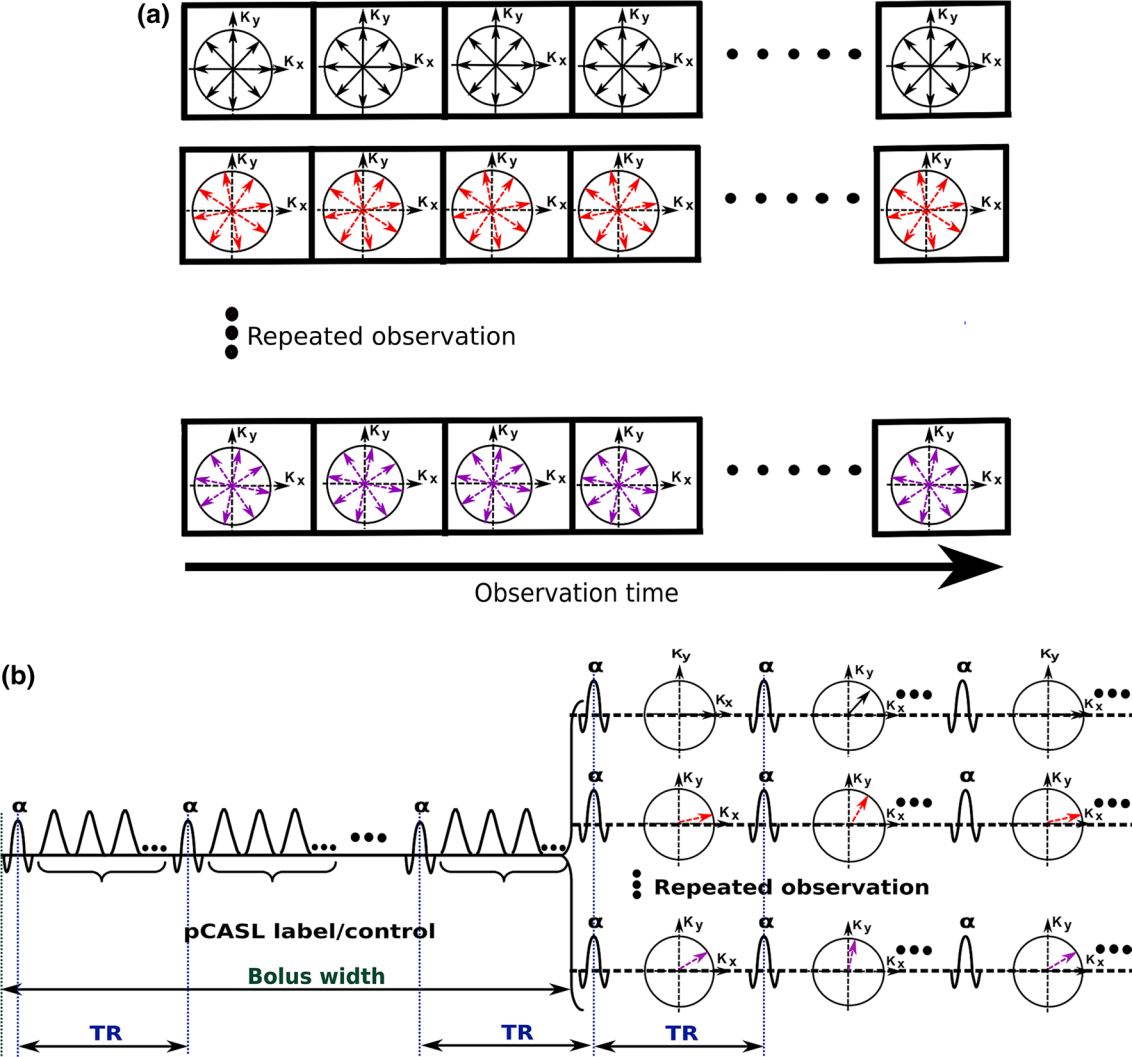
**a** Cine imaging of a periodic process starting with a block of multiple acquisitions of segment 1 (*first row*), followed by multiple acquisitions of segment 2 (*second row*), which is continued for all segments. If block onsets are synchronized to the periodicity of the process, all segments of the same column correspond to the same time point. Their combination yields densely sampled EPICYCLE data with a temporal resolution defined by the acquisition of a single segment. **b** Adaptation to bolus tracking by integration of pCASL before each block: pCASL preparation for the ‘label’ condition is followed by multiple acquisitions of segment 1, then the preparation is switched to the ‘control’, and the same block of segments is acquired. This scheme is repeated for all segments. The comb of Hanning RF pulses for pCASL is indicated by *underbraces*; it is periodically interrupted by slab-selective excitation of the image volume by pulses of flip angle *α* with a spacing defined by the repetition time ($T_{R}$) of the EPICYCLE module. Gradient pulses are not shown

To satisfy the Nyquist criterion, each spoke should consist of $n_{r} = N/2$ phase-encoding steps, and the number of spokes should be $n_{s} \ge \pi N$ for a $N \times N$ image matrix in the *xy*-plane [30]. Hence, the number of phase steps for EPICYCLE exceeds the number required for Cartesian 3D hybrid EPI by approximately 57 %. However, the cylindrical acquisition scheme offers a higher flexibility in the sampling order (Fig. 1c, d). In the current work, segmented sampling was employed as indicated in Fig. 1d [31]. It allows trading spatial resolution for temporal resolution in the observation of dynamic processes by reconstructing undersampled images from single segments [32]. Alternatively, the acquisition of segments can be arranged in blocks as shown in Fig. 2a to obtain a cine technique with an effective temporal resolution defined by the time required to sample all spokes of a single segment, $T_{\text{seg}} = \left( {n_{s} /n_{\text{seg}} } \right)T_{R}$ ($T_{R}$ is the repetition time). The temporal resolution is thus improved according to the number of segments, $n_{\text{seg}}$.

### Image acquisition

All experiments were carried out at 3 T on a MAGNETOM TIM Trio scanner (Siemens Healthcare, Erlangen, Germany) using the receive-only 32 channel head coil and the body transmit coil. The sequences were implemented under the Siemens IDEA environment and tested with a structural water phantom. A total of nine subjects (age 26.9 ± 4.8 years; five females) participated in this study.

EPICYCLE data were acquired with the readout axis along the head–foot direction and the phase blips in the perpendicular plane. Raw data at different spatial resolutions were obtained with $n_{z} = N/2 = 32, \, 48, \, 64,{\text{ or }}80$ readout samples; $n_{r} = N/2$; $n_{\text{seg}} = 16$; and $n_{s}$ rounded to an integer multiple of $n_{\text{seg}}$ close to $2\pi n_{r} /\varphi_{u}$, where $\varphi_{u} \ge 1$ is the undersampling factor (i.e., $\varphi_{u} = 1$ corresponds to full sampling). Images were reconstructed to a final matrix size of $N \times N \times N/2$ with a field of view (FOV) of 192 × 192 × 96 mm^3^, yielding nominal isotropic resolutions of Δ*x* = 3.0, 2.0, 1.5, or 1.2 mm, respectively. Other imaging parameters depending on the resolution are listed in Table 1. The reduced FOV in the head–foot readout dimension was achieved by selecting an axially oriented slab using a sinc-shaped RF pulse (duration 800 μs) that was approximately adjusted to the Ernst angle [33]; the delay for the slab-rephase gradient was 600 μs. In all cases, 150 dummy scans were applied prior to image acquisition.

**Table 1:** Acquisition parameters of EPICYCLE images in Fig. 3

Δ*x* (mm)	$$\begin{aligned} N = 2n_{r} \hfill \\ \quad = 2n_{z} \hfill \\ \end{aligned}$$	*n*_*s*_	Bandwidth (Hz/pixel)	*T*_*E*_ (ms)	*T*_*R*_ (ms)	*α* (°)
3.0	64	192	2694	1.8	45	15
2.0	96	288	1796	1.9	70	20
1.5	128	384	1346	2.0	100	25
1.2	160	480	1116	2.0	130	30

Bolus tracking experiments were performed with a nominal isotropic spatial resolution of 3.0 mm, $n_{z} = 32$, $n_{r} = 32$, a bandwidth of 3256 Hz/pixel, $\alpha$ between 4 and 16°, and *T*_*R*_ = 37 ms. With *n*_*s*_ = 96 spokes and *n*_seg_ = 32 or 48 segments; this yielded an effective temporal resolution of *T*_seg_ = 111 or 74 ms, respectively. By setting the number of acquisitions of the same segment, $N_{\text{acq}}$, to either 27 or 54, the duration of an acquisition block after pCASL preparation (i.e., the observation period of the ‘brain response curve’ within the imaging slab) was adjusted to 3 or 4 s, respectively.

For characterizing the distribution of resonance offsets, a $B_{0}$ map was acquired using a 3D multi-echo FLASH sequence with 12–24 bipolar gradient echoes, sagittal orientation with readout along the *z*-axis and the same slab dimensions and resolutions as used for EPICYCLE. The frequency offset in each voxel was obtained from a linear fit to the unwrapped phases of all unipolar echoes. The $B_{0}$ map was recorded with optimized shim settings achieved by multiple executions of the 3D shimming and frequency adjustment tools provided by the scanner software. Before starting the EPICYCLE scans, the 3D shim settings were copied from the preceding $B_{0}$ mapping scan to ensure that $B_{0}$ map and EPICYCLE data were acquired under identical conditions. This procedure was applied to all spatial resolutions separately.

### Image reconstruction

EPICYCLE image reconstruction was done offline using in-house software written in C++. The uncombined, raw *k*-space data in the proprietary Siemens TWIX format were used as input, and the reconstruction procedure was applied to every channel separately before combining the resulting single-channel images using the common sum-of-squares method.

After a one-dimensional Fourier transform in read-direction, the embedded template spoke was used to correct for Nyquist ghosting artifacts [24]. A linear fit of the unwrapped and thresholded phase differences between adjacent template lines was performed, and the resulting fit parameters were used to apply a phase correction to all pairs of adjacent lines within the spoke. The same template spoke was also used to correct all remaining spokes of the current volume. Finally, the phase differences of all central *k*-space lines were optionally used to correct for inter-spoke phase and intensity variations.

Following the phase correction, a resampling of the data in the *k*_*x*_ *k*_*y*_-plane onto an *N* × *N*, evenly spaced Cartesian grid ($N \equiv 2 n_{r}$ as defined above) was performed. The algorithm was based on O’Sullivan’s description [34] and was similar to the implementation by Hargreaves and Beatty [35]. After convolution with a Kaiser–Bessel kernel [36] with variable parameters to allow for optimization, a 2D fast Fourier transform was performed in the *k*_*x*_ *k*_*y*_-plane, followed by multiplication with an apodization-correction function [36, 37] to compensate for non-uniform *k*-space sampling density.

To correct for off-resonance effects, a multi-frequency approach was used [38–40]. Based on the separately acquired $B_{0}$ map, the range of off-resonance frequencies was segmented into a number of discrete, equally spaced frequencies, which could be further adjusted by an optional factor. The gridding procedure was subsequently performed for each of the selected frequencies. For the final image, those pixels from each reconstructed image whose $B_{0}$ values matched their reconstruction frequency were used.

### ASL-prepared EPICYCLE

The cine scheme shown in Fig. 2a was adapted such that a pCASL preparation module [26, 27] was applied immediately before each block of repetitive acquisitions of an individual *k*-space segment (Fig. 2b). The total duration of the pCASL module defining the width of a rectangular bolus of labeled blood was *τ* = 222 or 444 ms. As commonly done in ASL, each block of single segments was measured twice to collect separate data sets in the labeling and the control condition of pCASL. To maintain a steady state of the static tissue, the pCASL pulse comb was periodically interrupted for the excitation of the imaging slab by an RF pulse identical to that of the EPICYCLE readout. A non-selective fat saturation pulse combined with appropriate spoiler gradients was played out before each slab-selective excitation; however, only during the EPICYCLE module, but not during the pCASL preparation.

For pCASL, Hanning-shaped RF pulses (duration 500 μs, flip angle 22°, inter-pulse interval 1.4 ms) and a labeling gradient of 9 mT/m were used [26, 27]. Average values of the labeling RF amplitude and gradient were 1.05 μT and 0.6 mT/m, respectively. The phase increment between RF pulses was corrected for local magnetic field variations by introducing an additional phase increment [41]. Labeling was applied slightly inferior of the cerebellum [1].

### Evaluation of ASL bolus tracking

Raw data of all segments acquired in either the ‘label’ or ‘control’ condition were separately combined to yield a total *k*-space for each time point and reconstructed as described above. The time-dependent image volumes were then spatially smoothed using a 3D Gaussian filter (3-mm full width at half maximum, FWHM) and ‘control’ images were subtracted from the corresponding ‘label’ image to obtain a series of difference images with intensities $S_{L - C} \left( i \right)$, where *i* is the repetition index ($1 \le i \le N_{\text{acq}}$). Note that the pCASL preparation leads to a negative signal change in voxels affected by the bolus passage.

Quantitative characterization of the ASL bolus was based on in-house software written in IDL 8.1 (EXELIS Visual Information Solutions, Boulder, CO, USA). Consistent with previous evaluations of bolus dispersion [42], a gamma variate function,

1 $$h\left( {t_{s} } \right) = \Delta S \times \left( {t_{s} } \right)^{\sigma } \times \exp \left[ {\sigma \left( {1 - t_{s} } \right)} \right] \quad {\text{with}}\,\,t_{s} = \frac{{t - t_{0} }}{{t_{\text{peak}} - t_{0} }},$$

was employed as an empirical model function, where $t_{0}$ is the bolus arrival time, $t_{\text{peak}}$ is the time at which $h\left( {t_{s} } \right)$ is at maximum, and $\sigma$ is a shape parameter; $t$ denotes time and is obtained as $t = \left( {i - 1} \right) \times T_{\text{seg}}$. The signal amplitude is $\Delta S = S_{\hbox{max} } {-}S_{\hbox{min} }$ and is obtained by computing the minimum and maximum intensities according to $S_{ \hbox{min} } = { \hbox{min} }\left[ {S_{L - C} \left( i \right)} \right]$ and $S_{ \hbox{max} } = {\text{mean}}\left[ {S_{L - C} (i > 3N_{\text{acq}} /4)} \right]$, where ‘min’ and ‘mean’ denote the minimum and the mean functions, respectively. This base fitting function was applied to negative pCASL signal changes according to:

2 $$\tilde{h}\left( {t_{s} } \right) = S_{ \hbox{max} } - h\left( {t_{s} } \right).$$

The time to peak (TTP) measured from the center of an ideal bolus of duration *τ* is obtained as

3 $$\Delta t_{\text{peak}} = t_{\text{peak}} + \frac{\tau }{2}.$$

Least-squares fitting of $S_{L - C} \left( i \right)$ with σ, *t*_0_, and *t*_peak_ as free parameters was performed using the IDL function MPFITFUN.PRO, which employs a Levenberg–Marquardt algorithm [43, 44]. The range of the fitting parameters was restricted to $\sigma \ge 1$ and $t_{\text{peak}} \ge 0$. As $t = 0$ was defined as the time of the acquisition of the first segment (i.e., bolus generation occurred at negative times), $t_{0}$ was allowed to be negative in order to fit truncated bolus tracking curves. The FWHM of the fitted curve, $\Delta t_{1/2}$, was obtained by finding the two roots of the equation

4 $$h\left( {t_{s} + \frac{{t_{0} }}{{t_{\text{peak}} - t_{0} }}} \right) - \frac{1}{2} \times \Delta S \equiv 0$$

using the IDL function FX_ROOT.PRO, and by taking their difference. As an estimation of the goodness of the fit, the standard deviation of the residuals was calculated and expressed as percentage of the signal amplitude, Δ*S*. Typical values of the threshold for this standard error applied to the maps were 8–12 %.

## Results

### Basic EPICYCLE sequence

**Fig. 3:**
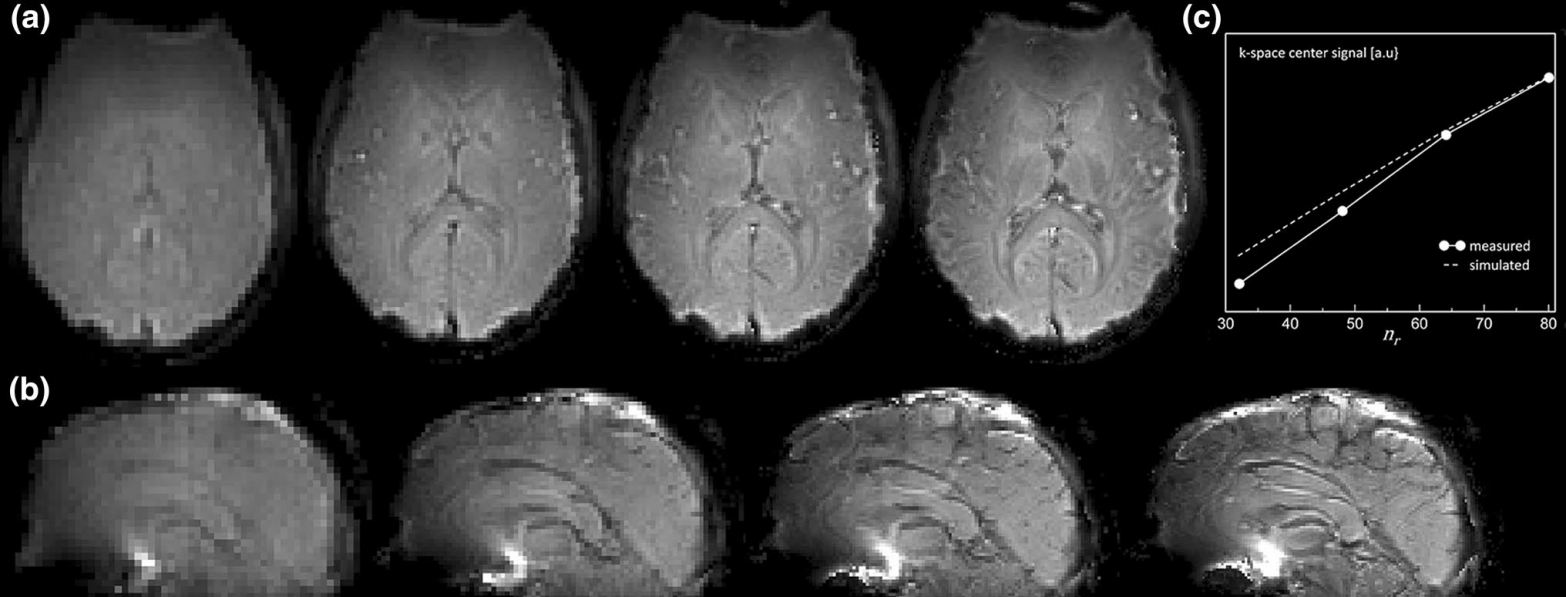
**a** Axial and **b** sagittal slices reconstructed from 3D EPICYCLE acquisitions with increasing nominal resolution (from *left* to *right*) of Δ*x* = 3.0, 2.0, 1.5, and 1.2 mm. **c** Plot of the measured signal amplitude in the center of *k*-space (*solid line*) versus the number of radial phase-encoding steps, $n_{r}$, and result from a simulation (*broken line*) based on Eq. (5), parameters from Table 1, as well as assumptions of *T*_1_ = 1 s and $T_{2}^{*}$ = 50 ms

In Fig. 3, axial and sagittal slices reconstructed from EPICYCLE acquisitions of different spatial resolutions are shown. All scans were recorded at the minimum possible $T_{E}$ and $T_{R}$ (Table 1). The observed increase of the signal intensity in the center of *k*-space, $s_{0}$, with increasing image resolution (Fig. 3c), is explained by the adaptations of $T_{R}$ (becomes longer with increased echo spacing) and, concomitantly, $\alpha$ (approximate Ernst angle assuming a longitudinal relaxation time, *T*_1_ ≈ 1 s; Table 1). Figure 3c further demonstrates that the change is consistent with the expected variation according to the Bloch equations [33]:

5 $$s_{0} \propto \frac{{1 - E_{1} }}{{1 - E_{1} \cos \alpha }}\left( {\sin \alpha } \right) e^{{ - T_{E} /T_{2}^{*} }} \quad {\text{with}} \; E_{1} \equiv e^{{ - T_{R} /T_{1} }} .$$

$T_{2}^{*}$ is the effective transverse relaxation time (assumed to be approx. 50 ms). Note that Eq. (5) solely holds for imaging of static tissue. The pCASL difference signal of arterial blood will be considered below.

Previously work has shown the occurrence of double contours in off-resonance regions on double-shot center-out EPI images due to the use of phase blips of opposite polarities [24]. In EPICYCLE, phase blips are rotated from shot to shot over a full circle, and the same off-resonance effect leads to multiple contours arranged as a ring pattern (Suppl. Fig. S1). These artifacts are effectively removed by using a multi-frequency image-reconstruction technique [38, 39].

As the $B_{0}$ map and the EPICYCLE scan are acquired with a temporal delay, the achieved image quality will be degraded if the field profile is not time invariant. Such inconsistency is expected for significant head motion during the scanning session as previously demonstrated [24]; this is a problem that is most straightforwardly addressed by implementing prospective motion correction [45]. A more subtle effect is obtained for $B_{0}$ drifts during the session. This leads to increased blurring in repeated EPICYCLE scans on images from later acquisitions, hinting on a progressive mismatch between the $B_{0}$ map recorded before the EPICYCLE series and the actual field pattern at the time of each individual scan. A correction of this effect based on the phase information from the central *k*-space lines is demonstrated in the Suppl. Fig. S2.

Visual inspection and evaluation of the signal-to-noise ratio of images reconstructed from the same acquisition upon progressively reducing the number of spokes indicated that twofold or threefold undersampling leads to a reasonable compromise between scan time and image quality (Suppl. Fig. S3). Previous work with radial encoding suggests that this limit may be pushed even further by additionally implementing parallel acquisition techniques and advanced reconstruction strategies [23].

### ASL bolus tracking

**Fig. 4:**
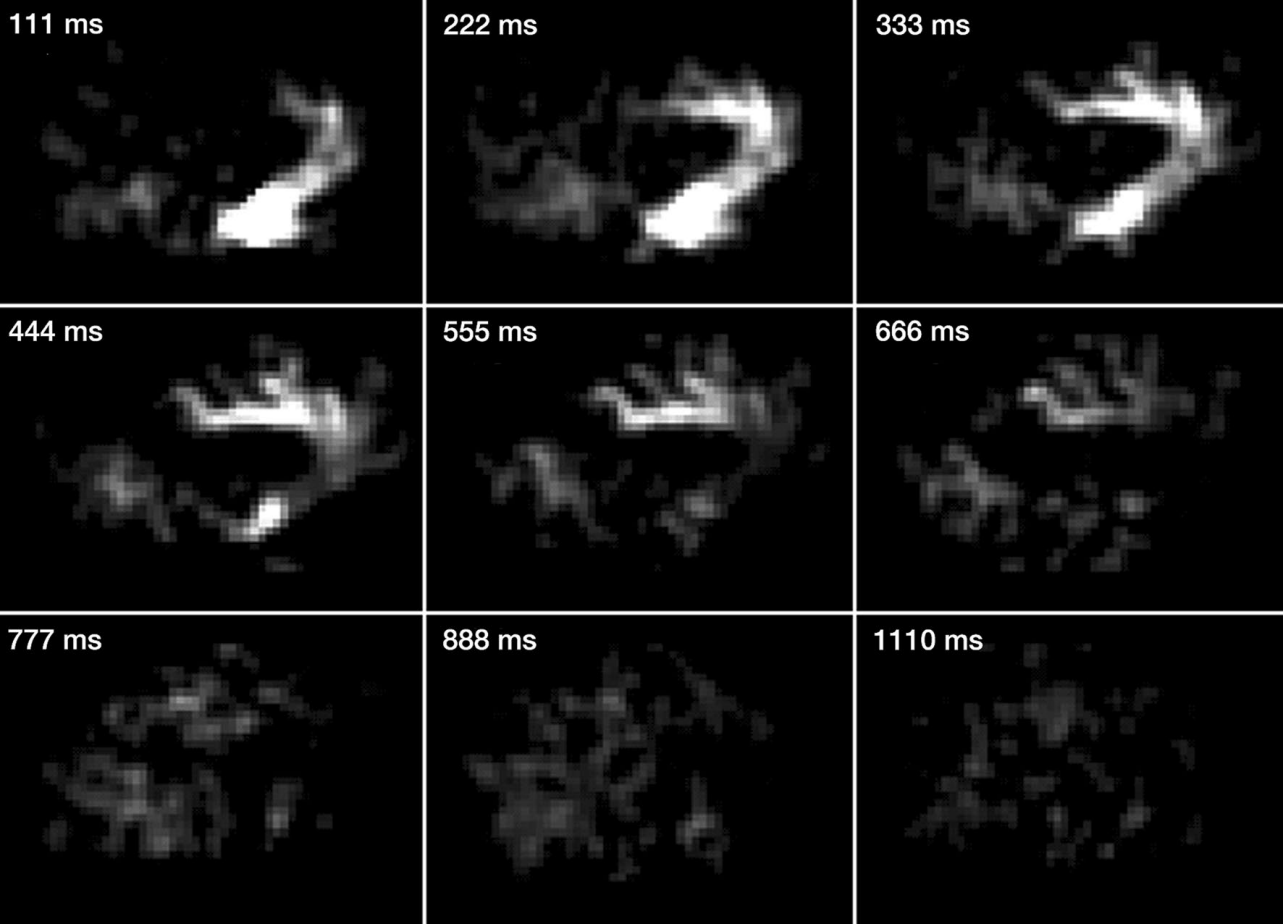
Highly contrasted ASL difference images (sagittal view of the medial brain) obtained with EPICYCLE (*α* = 4°) at successive time steps, demonstrating bolus passage through the branches of the anterior cerebral artery (ACA) with a time resolution corresponding to $T_{\text{seg}}$ = 111 ms. pCASL was used to generate a bolus ($\tau$ = 444 ms) of labeled arterial blood in a plane that was located 60 mm inferior from the magnet iso-center

Tracking of an ASL bolus generated with a pCASL module is demonstrated in Fig. 4. The segmented acquisition permits cine imaging of the transport of labeled blood from initial filling of larger-caliber arteries to subsequent dispersion in the downstream vasculature with a temporal resolution defined by $T_{\text{seg}}$. In this example, the distribution of the bolus in the branches of the anterior cerebral artery (ACA) is depicted. Due to the high blood velocity, a significant portion of the ACA is already visible at the first time step; hence, filling of the ACA with labeled blood had started during bolus generation by pCASL.

**Fig. 5:**
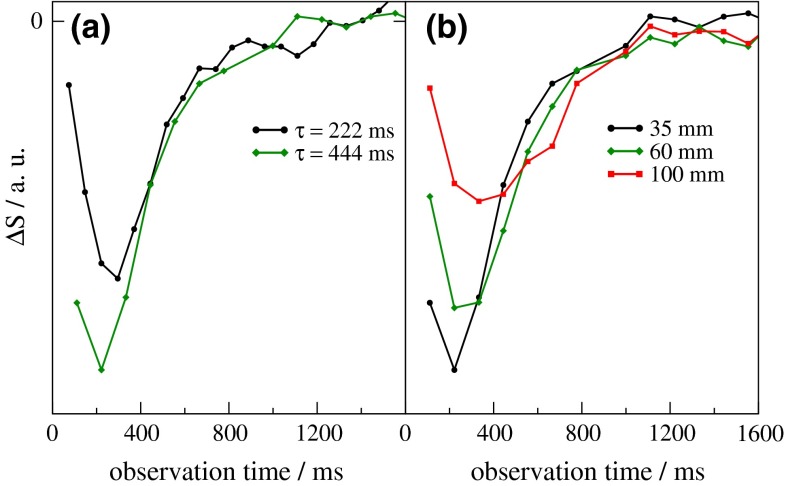
**a** ASL difference signal, $\Delta S_{L - C}$, in insular cortex acquired with *α* = 8° immediately after pCASL (35 mm inferior from the magnet isocenter) for labeling durations, $\tau$, of 222 (*black*) and 444 ms (*green*). **b** Time courses obtained in the same session with *α* = 8°, $\tau$ = 444 ms, and with increasing distances (hence, correspondingly increasing ATT) of 35 mm (*black*), 60 mm (*green*), and 100 mm (*red*) between labeling plane and magnet isocenter

To investigate the consistency of such experiments, the influence of parameters affecting the bolus shape (i.e., $\tau$ and the labeling plane position) and of the imaging RF pulses were systematically studied. Results obtained from a small region of interest (ROI) in the left insular cortex are presented in Fig. 5. If $\tau$ is shorter than the ATT from the labeling plane to the inferior edge of the imaging slab, both a raising and a falling edge of the ‘brain response curve’ are captured during the acquisition window. Note that we use the term ‘brain response curve’ for a bolus shape measured in a voxel of the imaging slab to better distinguish it from the (ideal) bolus shape generated by pCASL at the labeling plane. Figure 5a demonstrates that the signal response of a longer ASL bolus appears truncated, whereas a shorter bolus is almost entirely recorded. Besides shortening the pCASL bolus duration, truncation of the recorded ‘brain response curve’ can also be reduced by increasing the distance between the labeling plane and the imaging slab. This is demonstrated in Fig. 5b, where the peak minimum appears shifted towards a later observation time with increased distance. Simultaneously, the FWHM of the ‘brain response curve’ increases with distance, which is caused by an enhanced dispersion of the arterial arrival times. Finally, the more intense tail obtained with the selection of a distance of 100 mm is consistent with the assumption of laminar flow.

**Fig. 6:**
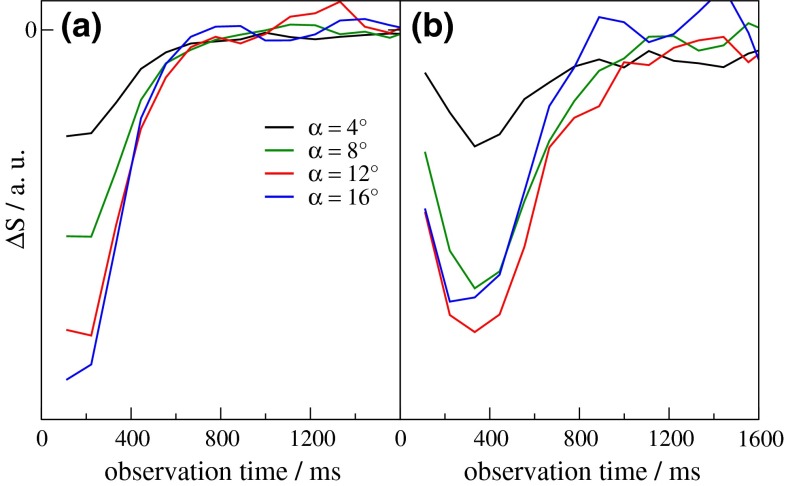
Time courses of the ASL difference signal, $\Delta S_{L - C}$, for variable flip angles, $\alpha$, of the EPICYCLE readout as indicated. Data were taken in the same session with $\tau$ = 444 ms from **a** an upstream ROI near the circle of Willis and **b** a supraventricular downstream ROI in the ACA territory. The distance between labeling plane and magnet isocenter was 60 mm

The attenuation of the ASL difference signal by repetitive RF irradiation depends on the number of pulses, $n_{p}$, experienced by the flowing blood [5]. Because the signal amplitude is proportional to $({ \cos }\; \alpha )^{{n_{p} - 1}} \sin \alpha$, the maximum signal is obtained for a flip angle that obeys the relation $\tan \alpha_{\text{opt}} = \left( {n_{p} - 1} \right)^{ - 1/2}$. This yields optimum angles of 90, 30, 18.4, and 9.1° for examples of *n*_*p*_ = 1, 4, 10, and 40, respectively. As *n*_*p*_ increases with the ATT (here measured as $t_{\text{peak}}$), $\alpha_{\text{opt}}$ concomitantly decreases and can be estimated using $n_{p} = t_{\text{peak}} /T_{R}$. This behavior was confirmed experimentally by comparing ASL difference signals at upstream and more downstream positions and variation of α (Fig. 6). The largest upstream signal difference (besides stronger bolus truncation due to shorter ATT) is obtained with *α* = 16° (Fig. 6a); whereas, more downstream, *α*_opt_ ≈ 12° was found (Fig. 6b). Thus, the choice of *α* determines the sensitivity of the sequence to smaller downstream arteries, and the penetration depth of the bolus in the imaging slab can be improved by choosing moderate flip angles between 8 and 12°.

**Fig. 7:**
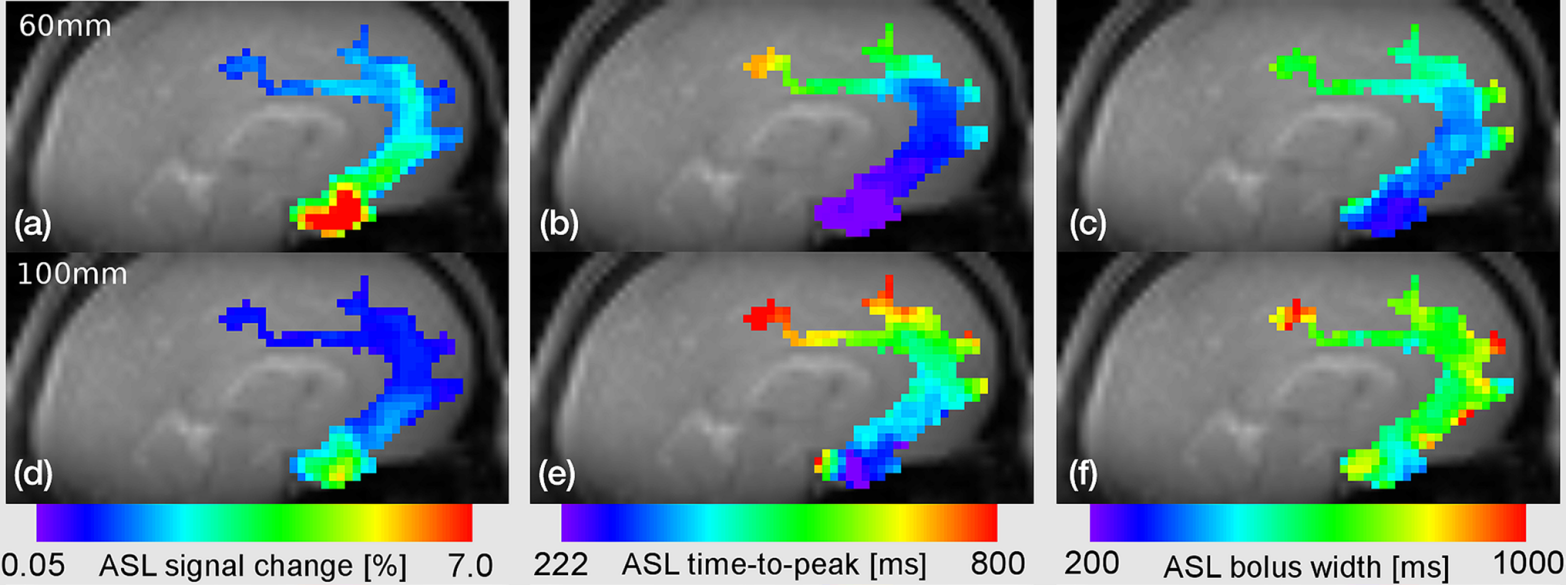
*Color-coded* maps of **a**, **d** the relative ASL signal change, **b**, **e** $\Delta t_{\text{peak}}$, and **c**, **f** $\Delta t_{1/2}$ from a ROI of the left ACA obtained by fitting the ASL difference signal from EPICYCLE bolus tracking experiments with $\tau$ = 444 ms, *α* = 8°, and distances between the labeling plane and the magnet isocenter of **a**–**c** 60 and **d**–**f** 100 mm

Parameter maps of Δ*S*, Δ*t*_peak_, and $\Delta t_{1/2}$ obtained from voxel-by-voxel fits of the ‘brain response curves’ to Eq. (2) are depicted in Fig. 7. In particular, the region of the ACA ranging from the circle of Willis to the supraventricular brain is shown for different distances between the labeling plane and the magnet isocenter. The parameter maps reflect expected features of CBF: (1) the largest signal change is observed near the circle of Willis with more prominent values along the ACA main branch (Fig. 7a, d) or at positions where the middle cerebral artery perforates the insular region (not shown). (2) The TTP increases quite uniformly for more downstream positions (Fig. 7b, e). This quantity, however, was truncated for voxels near the circle of Willis, because only the falling edge of the ‘brain response curve’ was observed in this region due to the fast blood transport [5]. Note that $\Delta t_{\text{peak}} \ge \tau /2$ according to Eq. (3), that is, the minimal TTP that can be detected (for the hypothetical case of no transit delay) is defined by half the duration of the pCASL module, indicating that relatively short labeling durations are attractive for studying more proximal vessel segments. (3) Similar to the TTP, the FWHM increases along the vessels moving downstream (Fig. 7c, f). By comparing maps from experiments with different positions of the labeling plane at the same downstream positions, the influence of these parameters can be assessed directly. As expected, for increasing distance between the inferior edge of the slice package and the labeling plane, the signal amplitudes decrease while the TTP and FWHM increase (top and bottom rows in Fig. 7).

**Fig. 8:**
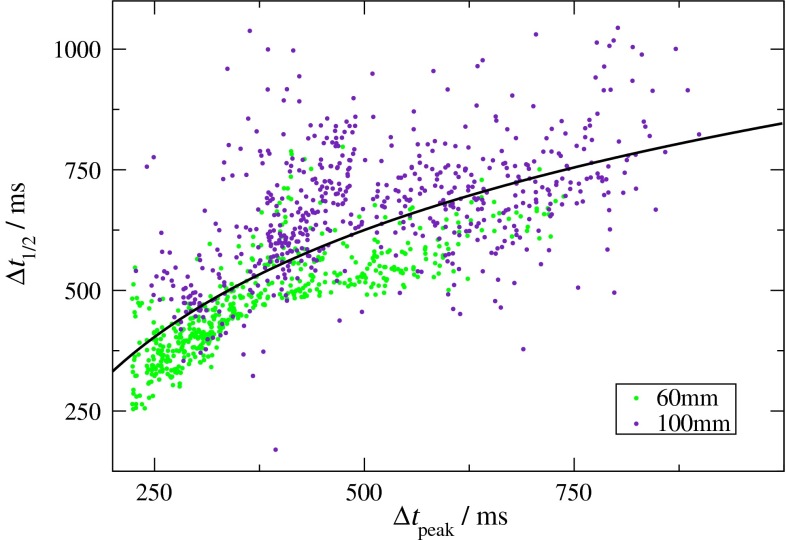
*Scatter plot* of $\Delta t_{1/2}$ versus $\Delta t_{\text{peak}}$ from an ROI in the left ACA for bolus-tracking experiments in the same subject with *τ* = 444 ms and *α* = 8°. Data were obtained with distances between the labeling plane and the magnet isocenter of 60 (*green dots*) and 100 mm (*indigo dots*). Voxels with $\Delta t_{\text{peak}} = \tau /2$ were excluded to remove effects from ASL bolus truncation. The *solid trend line* is inserted as a guide to the eye to indicate the sublinear dependence of $\Delta t_{1/2}$ on $\Delta t_{\text{peak}}$

The relation between $\Delta t_{1/2}$ and $\Delta t_{\text{peak}}$ can be assessed from scatter plots of both quantities as shown in Fig. 8. The data were obtained in the ACA territory for distances of 60 and 100 mm between the labeling plane and the magnet isocenter. A visual inspection of the points suggests sublinear behavior; that is, the slope of $\Delta t_{1/2}$ progressively declines with $\Delta t_{\text{peak}}$.

## Discussion

In this work, EPICYCLE was introduced as a novel readout for fast 3D imaging of the brain, which combines features from center-out EPI with those from radial encoding by rotating the phase-blip gradients from shot to shot, and thus obtaining a cylindrical *k*-space volume. A drawback related to this approach that yields a short $T_{E}$, is the occurrence of ring-like contours in case of image distortions caused by inhomogeneity of $B_{0}$. Therefore, the achieved image quality heavily depended on the accuracy of the $B_{0}$ map employed for artifact correction during image reconstruction. In practice, mainly $B_{0}$ drifts during the separate acquisition of the $B_{0}$ map as well as during the EPICYCLE time series cause blurring of the EPICYCLE images, especially at higher resolution. A reduction in scan time for the $B_{0}$ map by parallel imaging techniques and the drift correction of the EPICYCLE time series (Suppl. Fig. S2) are strategies to mitigate this problem.

It should be noted that previous cylindrically encoded 3D EPI variants have been successfully applied to diffusion-weighted imaging [10] or bolus tracking in DSC-MRI [11]. The outcome of these variants is less dependent on a $B_{0}$ map because, therein, phase-blips are kept constant, and the readout gradient is rotated from shot to shot. Future work is needed to explore the most favorable sequence design. This should be expanded to include further modifications of the existing implementation, such as a possible omission of the slab-selective excitation during the pCASL labeling module.

Gamma variate functions as employed in the current study to fit single-voxel ‘brain response curves’ have been extensively used in DSC-MRI (e.g., [46]) to obtain a parametric representation of the residue function and to estimate cerebral perfusion. This choice, however, appears rather empirical and may not fully account for relaxation and signal attenuation from repetitive RF pulsing. A recent kinetic model for vessel-encoded dynamic MRA [5] includes these effects explicitly in an ASL approach. The experimental conditions present in our study, however, differ from previous ones [5]. Firstly, instead of imaging a single (thick) slice, we evaluated a 3D slab, which implies that bolus dispersion during passage through the slab is not negligible. Secondly, shorter ASL bolus durations enabled the observation of the raising and falling edges of the vascular ‘brain response curves’ in most imaging voxels. Thirdly, imaging RF pulses were applied also during the pCASL module to maintain a steady-state signal from static tissue. In order to assess the impact of the imaging pulses on the observed ‘brain response curves’, we used the above stated model [5] for conducting additional simulations (Suppl. Fig. S4). They show that for conditions of short ASL bolus durations and continuing application of imaging pulses as in our study, distinct features in the observed ‘brain response curves’ are not expected. Instead, the overall effect of the imaging RF pulses is rather a moderate scaling of the undisturbed ‘brain response curves’ (Suppl. Fig. S4b, solid lines). Hence, the curves obtained with the current range of parameters seem to lack sufficient specificity for allowing reliable fits to more elaborate biophysical models.

Another aspect is that the adapted model [5] used for simulations (Suppl. Fig. S4) does not account for bolus dispersion during transit from the labeling plane to the imaging region. A more rigorous description of ASL bolus tracking would be obtained by considering the evolution of the ASL bolus from an ideal rectangular shape at the labeling plane to the observed ‘brain response curve’ in a specific imaging voxel that reflects the local AIF. Previous work has shown that the arterial vascular structure is self similar and follows Murray’s law [47, 48]. This permits to approximate the underlying process by a convolution chain of individual transport functions for each vessel generation [49]. A transport function suitable to describe the passage of a contrast agent (gadobutrol) through the vascular tree has been recently suggested [49]. It follows from the assumption of laminar flow and decays quadratically with time yielding—upon application of the convolution chain—a linear dependence of $\Delta t_{1/2}$ on $\Delta t_{\text{peak}}$; that is, a progressive broadening of the AIF. At first sight, the sublinear dependence of $\Delta t_{1/2}$ on $\Delta t_{\text{peak}}$ observed in the current study (Fig. 8) seems to contradict this model. However, this apparent discrepancy can be attributed to the different nature of the tracers used in ASL and DSC-MRI. In particular, the ASL bolus undergoes $T_{1}$ relaxation during transit, which results in a superimposed exponential decay with time, and hence, a sublinear dependence of $\Delta t_{1/2}$ on $\Delta t_{\text{peak}}$. These considerations should be further investigated on a quantitative level in future work.

## Conclusion

EPICYCLE was introduced as a novel 3D EPI readout with cylindrical center-out spatial encoding. For bolus tracking, a segmented cine acquisition scheme and preceding short periods of pCASL were combined in a way that preserved the steady state of the tissue in the 3D imaging slab. Tracking of the thereby created pCASL bolus with temporal resolutions in the order of 100 ms and successive empirical modeling yielded parametric maps for a characterization of cerebral blood transport and arterial dispersion in large vessels.

## Electronic supplementary material

Below is the link to the electronic supplementary material.

Supplementary material 1 (PDF 1587 kb)
