# Supplementary material for: Three-dimensional echo-planar cine imaging of cerebral blood supply using arterial spin labeling
Source: MAGMA. 2016 May 25;29(6):799–810. doi: 10.1007/s10334-016-0565-0 (PMC5124058; doi:10.1007/s10334-016-0565-0)
Supplement: Supplementary file 1 — Supplementary material 1 (PDF 1587 kb) [file 10334_2016_565_MOESM1_ESM.pdf]

Electronic Supplementary Material

Three-Dimensional Echo-Planar Cine Imaging of Cerebral Blood Supply Using Arterial Spin Labeling

Manoj Shrestha[1*] • Toralf Mildner[1*] • Torsten Schlumm[1*] • Scott H. Robertson[2] • Harald E. Möller[1]

[1]*Max Planck Institute for Human Cognitive and Brain Sciences, Leipzig, Germany*
[2]*Center for In Vivo Microscopy, Duke University Medical Center, Durham, NC, USA*

Abbreviations

2D, 3D = two-, three-dimensional; ACA = anterior cerebral artery; AIF = arterial input function; ASL = arterial spin labeling; ATT = arterial transit time; CBF = cerebral blood flow; CV = coefficient of variation; DSC = dynamic susceptibility contrast; EPI = echo-planar imaging; EPICYCLE = echo-planar imaging with cylindrical center-out spatial encoding; FLASH = fast low-angle shot; FOV = field of view; FWHM = full width at half maximum; MRA = magnetic resonance angiography; MRI = magnetic resonance imaging; pCASL = pseudo-continuous arterial spin labeling; RF = radiofrequency; ROI = region of interest; TTP = time to peak.

Mathematical Symbols

B_0: main magnetic field amplitude,
E_1: longitudinal relaxation factor,
$h(t_S)$: gamma variate function,
$\tilde{h}(t_S)$: gamma variate function as applied to negative signal changes,
i: repetition index,
k_x, k_y, k_z: k-space coordinates,
N: (quadratic) image matrix size,
N_{acq}: number of acquisitions of identical segments,
n_p: number of RF pulses,
n_r: number of sampling points along a radius in k-space,
n_s: number of spokes,
n_{seg}: number of segments,
n_z: number of sampling points along readout direction in k-space,

---

* These authors contributed equally to this work.

S_{L-C}: pixel signal intensity in difference images of 'label' minus 'control' condition,
S_{max}: maximum signal intensity,
S_{min}: minimum signal intensity,
s_0: signal intensity in the center of k-space,
ΔS: signal amplitude,
T_1: longitudinal relaxation time,
T_2^*: effective transverse relaxation time,
T_E: echo time,
T_R: repetition time,
T_{seg}: acquisition time for a single segment,
t: time,
t_0: arrival time parameter of the gamma variate function,
$\Delta t_{1/2}$: FWHM of the gamma variate function,
t_{peak} time at which the gamma variate function is at maximum,
Δt_{peak} time to peak from the center of the bolus at the labeling plane,
t_s: dimensionless argument of the gamma variate function obtained by shifting and scaling of t,
x, y, z: Cartesian coordinates,
Δx: nominal image resolution (in x-direction)
α: RF pulse flip angle,
γ: gyromagnetic ratio,
σ: shape parameter of the gamma variate function,
τ: duration of the pCASL module (i.e., ideal bolus width),
φ_u: undersampling factor.

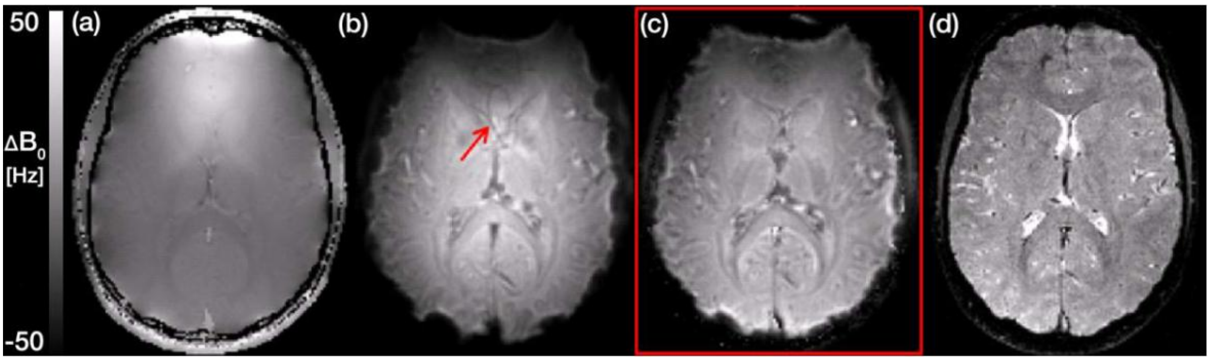

Supplementary Figure S1. Axial slices reconstructed from an EPICYCLE acquisition to illustrate the correction of distortions due to B_0 inhomogeneity. **(a)** Corresponding slice from a separately recorded B_0 map; **(b)** uncorrected EPICYCLE slice demonstrating ring-like contours in regions with significant offset from resonance (red arrow); **(c)** the same slice after distortion correction; **(d)** corresponding slice from an acquisition with 3D GRE imaging with Fourier encoding for comparison as an undistorted reference.

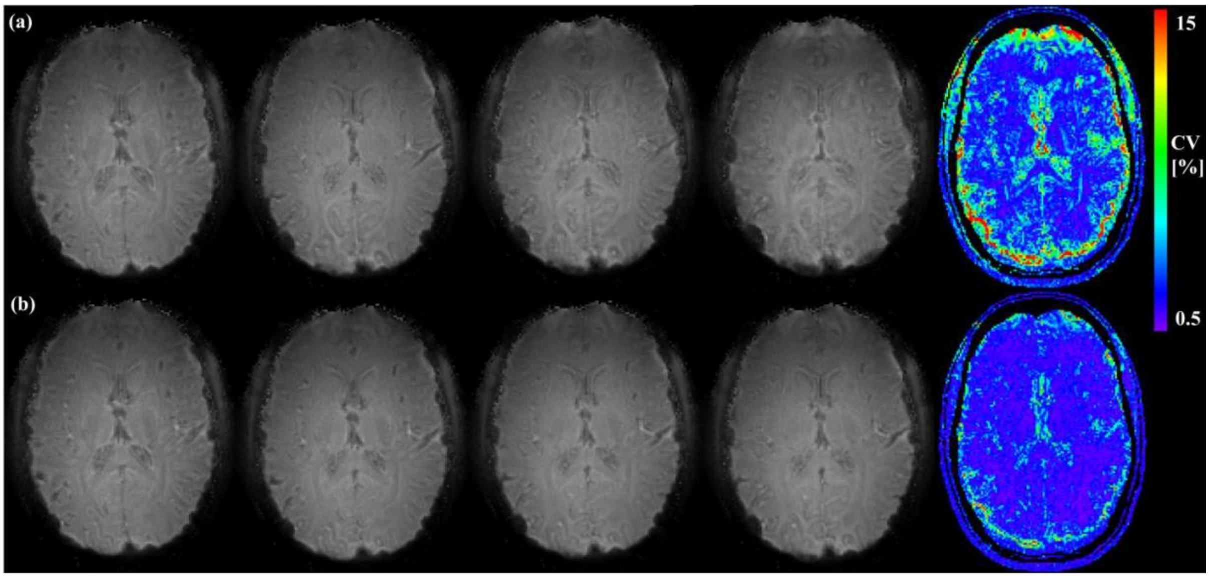

Supplementary Figure S2. (a) Axial slices from four consecutive 3D EPICYCLE data sets obtained with multi-frequency reconstructions based on the information from a previously acquired single B_0 map. During the time required for the EPICYCLE scans (10:44 min overall), a global drift of the main magnetic field corresponding to $\gamma B_0/(2\pi) \approx 10$ Hz was observed leading to progressive degradation of the distortion correction (from left to right). **(b)** The same images obtained with additional correction of the field drift. In particular, the evolution of the mean phase of the central k-space line that is repetitively acquired with every spoke allows tracking of long-term global B_0 drifts. The phases of all central lines were, thus, averaged for each repetition to calculate a repetition-specific correction offset for the B_0 map used during the multi-frequency step, which achieved a relevant visible improvement of image blurring. The right column shows color-coded maps of the coefficient of variation (CV) of the image series demonstrating a substantial reduction after the drift correction.

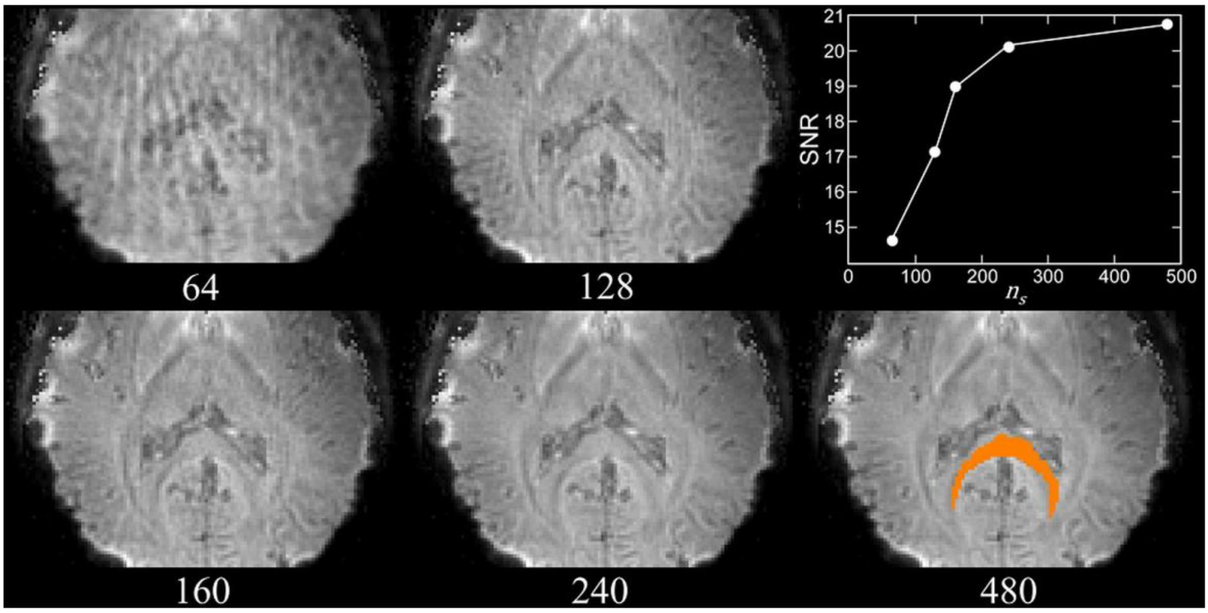

Supplementary Figure S3. Examples of EPICYCLE images of the same subject obtained with different undersampling factors, φ_u= 7.5, 3.75, 3, 2, and 1. All images were reconstructed to the same matrix with nominal isotropic resolution of 1.2 mm. The graph shows average signal in the *corpus callosum* (ROI indicated in orange) normalized by the SD as a function of the number of spokes (n_s = 64, 128, 160, 240, and 480 as indicated below the images).

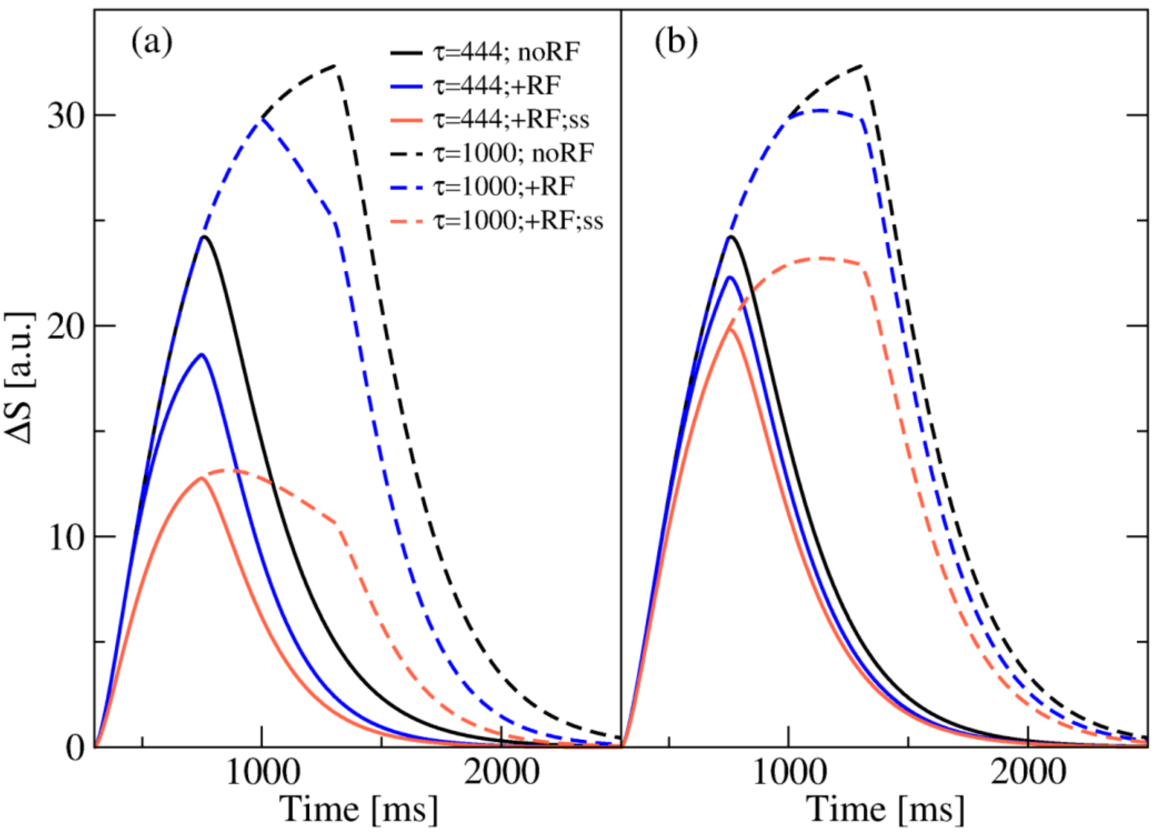

Supplementary Figure S4. Influence of imaging RF pulses on the time-dependent ASL signal difference resulting from an ASL bolus of length τ of 444 or 1000 ms as indicated. Assumed repetition times are **(a)** T_R = 18 ms and **(b)** T_R = 37 ms with corresponding flip angles of 10° and 8°, respectively. For each case, three curves are shown, namely, without any imaging RF pulses ('noRF'), with the RF pulses starting after cessation of the pCASL module ('+RF'), and with the RF pulses applied simultaneously with the pCASL module ('+RF;ss'), that is, the pCASL module was periodically interrupted for excitation of the imaging slab. The curves were obtained by adaptation of the kinetic model of Okell *et al.* [S1] with the following parameters: A = 50, p = 150 ms, s = 0.004 ms^{-1}, δt = 300 ms, and δt_{min} = 100 ms. The parameters p and s characterize the underlying gamma variate dispersion kernel, δt and δt_{min} denote the blood transit times to the imaging voxel and to the lower edge of the imaging slab, respectively, and A is a scaling factor [S1]. Note that full vascular 'brain response curves' are presented in these graphs, whereas experimentally obtained curves are truncated, that is, ASL signal changes are not measured during execution of the pCASL module of duration τ that precedes the imaging module.

Reference

S1. Okell TW, Chappell MA, Schulz UG, Jezzard P (2012) A kinetic model for vessel-encoded dynamic angiography with arterial spin labeling. Magn Reson Med 68:969-979.
